# Research progress on exercise fatigue from the perspective of fatigue biomarkers

**DOI:** 10.7717/peerj.20424

**Published:** 2026-01-07

**Authors:** Xin Liu, Juan Liu

**Affiliations:** 1 Beijing Key Laboratory of Diagnostic and Traceability Technologies for Food Poisoning, Beijing Center for Disease Prevention and Control, Beijing, China; 2 Biology and Agriculture Research Center, University of Science and Technology Beijing, Beijing, China

**Keywords:** Exercise fatigue, Central fatigue, Peripheral fatigue, Biomarkers

## Abstract

Exercise-induced fatigue refers to the physiological processes of body functions that cannot be sustained at a specific level during exercise or the inability of the organs to maintain a predetermined level of intensity. Exercise-induced fatigue is a comprehensive physiological process, which is mainly reflected in the body’s neuromuscular system and cardiovascular system. The study of fatigue-related physiological responses related to exercise-induced fatigue provides crucial insights into the underlying mechanisms, enables the assessment of fatigue levels, and aids in the formulation of effective recovery strategies. This review summarized the latest advancements in the research of biomarkers associated with exercise-induced fatigue, exploring the mechanisms of various biomarkers, detection methods, and their applications in sports medicine. Studies have shown that energy substances, metabolites, blood bioindicators, central neurotransmitters, free radicals, urine, saliva, etc., are related to exercise-induced fatigue-related biomarkers in human body. Among them, energy-related substances were the first fatigue markers studied, and metabolites in blood or urine were gradually used as biomarkers as research was deepened and testing methods were refined. The presence of central neurotransmitters gradually increased, and researchers gradually emphasized the important role of neurotransmitters in exercise-induced fatigue. Through a comprehensive analysis of relevant literature, this paper aimed to offer guidance for future research directions and promote a more scientific approach to managing exercise-induced fatigue.

## Introduction

Exercise-induced fatigue can be caused by central nervous system abnormalities, namely central fatigue, or peripheral nervous system disorders (Hsiao et al., 2018). Exercise-inducedfatigue is a multifaceted physiological phenomenon that arises from the intricate interplay between various systems, including the nervous, muscular, and metabolic systems (Song et al., 2023). At present, many theories have been proposed on the possible mechanism of exercise-induced fatigue, mainly including energy depletion theory (Noakes, 2000), metabolite accumulation theory (Liu et al., 2022; Zhao et al., 2023), free radical theory (Chen & Liu, 2022; Dobryakova et al., 2015; Ronghui, 2015), internal environment homeostasis disorder theory (Ament & Verkerke, 2009), fatigue chain theory (Girard, 2014), central nervous system transmitter imbalance theory, protective inhibition theory, mutation theory, *etc*., but the mechanism has not been fully elucidated; the identification and understanding of biological markers of exercise-induced fatigue have become increasingly important. Biomarkers are biochemical markers that can mark changes in structure or function from a system to cells or even subcell (Califf, 2018). Understanding the biomarkers of exercise-induced fatigue can be beneficial to understand the grading of exercise-induced fatigue, and it is also of guiding value for subsequent therapeutic recovery. This review aims to systematically examine the current research advancements regarding biomarkers of exercise-induced fatigue, analyze their biological underpinnings, and explore their potential clinical applications.

This paper presents a narrative review of the latest research progress on exercise-induced fatigue biomarkers, filling the knowledge gap in the interdisciplinary field and pssessing both academic and applied value. This article incorporates markers of different dimensions, such as energy metabolism, metabolites, neurotransmitters, and free radical damage, into a unified framework to elucidate the multi-system interaction mechanism of exercise-induced fatigue and provide theoretical support for interdisciplinary research. It disscusses emerging detection technologies, such as microneedle sensors and salivary metabolomics, which promote the development of non-invasive, real-time monitoring devices and address the limitations of traditional blood tests. The article emphasizes the practical value of biomarkers in optimising training programmes, preventing sports injuries, and managing chronic fatigue, thereby providing a foundation for scientific intervention. The direction of multi-omics integration, standardised testing, individualised intervention, *etc*. is proposed to show the breakthrough pathway for both academia and industry.

The target audience for this article includes exercise science researchers, clinical and sports medicine practitioners, and sports practitioners who can learn about the latest research progress and mechanism analysis of fatigue markers, how to optimise exercise performance and recovery through plant-based nutrition, and how to adjust training intensity *via* real-time lactate monitoring. In addition, practitioners in the food and nutrition industry can use this article to understand market demands and trends in research and development, and develop wearable devices or anti-fatigue products with reference to technological trends. By integrating multidisciplinary perspectives, this article aims to provide both scientific depth and practical value to different groups of readers.

## Survey Methodology

A comprehensive literature search was conducted using the PubMed, Google Scholar, SPORTDiscus, and Web of Science databases. The initial search was performed on February 6, 2025, with the final search completed on August 25, 2025. The search strategy incorporated the following key terms and combinations: (1) the phrase “exercise fatigue” was combined with “mechanism”, “physical”, “central”, “peripheral” to locate studies related to the mechanisms of exercise-induced fatigue; (2) the term “fatigue biomarker” was paired with “metabolism”, “metabolites”, “blood”, “non-invasive”, “urine”, “urinary”, “saliva”, to ensure the retrieval covered different categories of biomarkers; (3) the terms “exercise,” “fatigue,” and “biomarker” were used in conjunction with “monitor,” “detection,” “microneedle sensor,” “biochip,” or “mass spectrometry” to incorporate methodological advances in biomarker quantification and application. While emphasis was placed on recent publications, seminal earlier works were also considered where appropriate. Retrieved records were initially screened for relevance based on title and abstract. Articles meeting the inclusion criteria underwent full-text review and detailed evaluation. Finally, this review identified a total of 115 pertinent articles.

## Physiological Mechanisms of Exercise-Induced Fatigue

Exercise-induced fatigue can be classified into two primary categories: central fatigue and peripheral fatigue (Chen & Liu, 2022). Central fatigue originates from the central nervous system (CNS), impacting the brain’s ability to initiate and sustain motor output. It is characterized by a reduction in voluntary muscle activation, often associated with psychological factors such as motivation and perceived exertion (Bassett, 2000). In contrast, peripheral fatigue occurs at the muscle itself, arising from biochemical changes in muscle fibers during prolonged exercise. It includes the depletion of energy substrates, accumulation of metabolic byproducts like lactate, and alterations in ion concentrations that lead to impaired muscle contractility (Allen, Lamb & Westerblad, 2008; Lei et al., 2024). Studies have shown that central fatigue can be exacerbated by mental fatigue, which influences neurotransmitter levels and alters the perception of effort during physical activity (Baek et al., 2024; Meeusen, Van Cutsem & Roelands, 2021; Nybo & Secher, 2004; Telles et al., 2023). Understanding these distinctions is crucial for developing targeted interventions to mitigate fatigue and enhance athletic performance (Domínguez et al., 2022; Hennessy, 2022).

**Figure 1:**
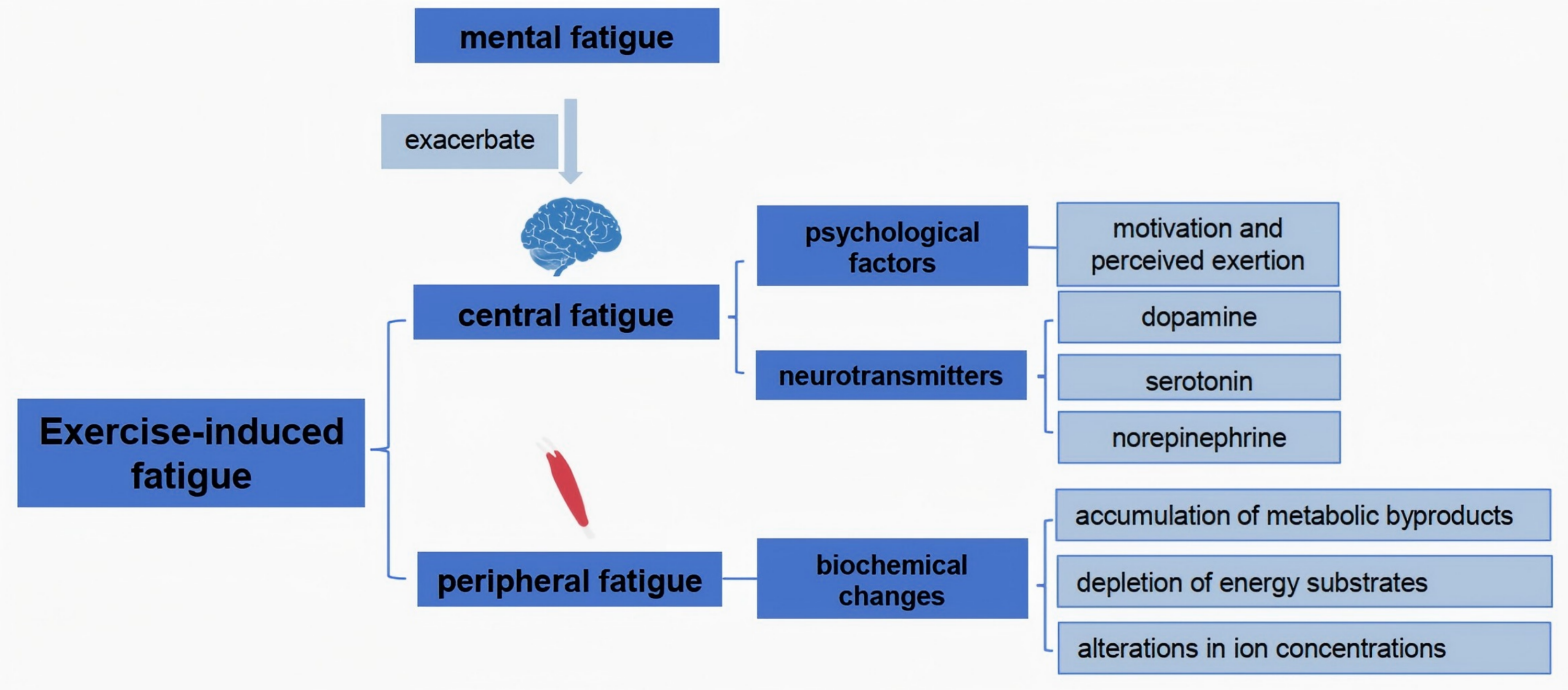
Mechanistic analysis of exercise fatigue.

Neurotransmitters play a pivotal role in the onset and progression of exercise-induced fatigue. During physical exertion, neurotransmitters such as dopamine, serotonin, and norepinephrine are involved in regulating mood, motivation, and motor function (Ament & Verkerke, 2009). Research indicates that the noradrenergic system, in particular, is linked to central fatigue, as increased norepinephrine levels correlate with heightened perceptions of exertion (Meeusen, Van Cutsem & Roelands, 2021). Additionally, serotonin is involved in the fatigue response, as alterations in its levels can influence both physical performance and mental fatigue (Falabrègue et al., 2021). The balance of these neurotransmitters is critical; for instance, low levels of serotonin are associated with increased fatigue and depressive symptoms, suggesting that interventions aimed at optimizing neurotransmitter levels could mitigate fatigue and enhance performance (Ma et al., 2021). Furthermore, the interplay between neurotransmitter systems highlights the complex nature of fatigue, encompassing both physiological and psychological dimensions (Fig. 1).

Metabolic byproducts, particularly those produced during high-intensity exercise, significantly affect muscle function and contribute to fatigue. The accumulation of lactate and hydrogen ions lowers the pH within muscle cells, impairing enzymatic activity and disrupting calcium handling, which is essential for muscle contraction (Tornero-Aguilera et al., 2022). Additionally, the depletion of glycogen stores during prolonged exercise can limit energy availability, further exacerbating fatigue (Mast et al., 2025). Studies have shown that strategies to enhance recovery, such as carbohydrate supplementation and proper hydration, can mitigate the effects of metabolic byproducts and improve performance outcomes (Keller et al., 2021). Understanding the biochemical pathways involved in fatigue can inform training regimens and recovery protocols, ultimately leading to improved athletic performance and a reduced risk of overtraining or injury.

## Classification and Characteristics of Major Biomarkers

### Substances related to energy metabolism

The onset and progression of exercise-induced fatigue are intrinsically linked to the body’s capacity to generate and regulate energy. Within this context, the depletion, accumulation, and metabolic flux of specific energy substrates and their related compounds serve as critical physiological indicators. Consequently, substances directly involved in cellular energy pathways constitute a primary category of biomarkers for assessing fatigue.

#### Adenosine triphosphate and creatine phosphate

**Figure 2:**
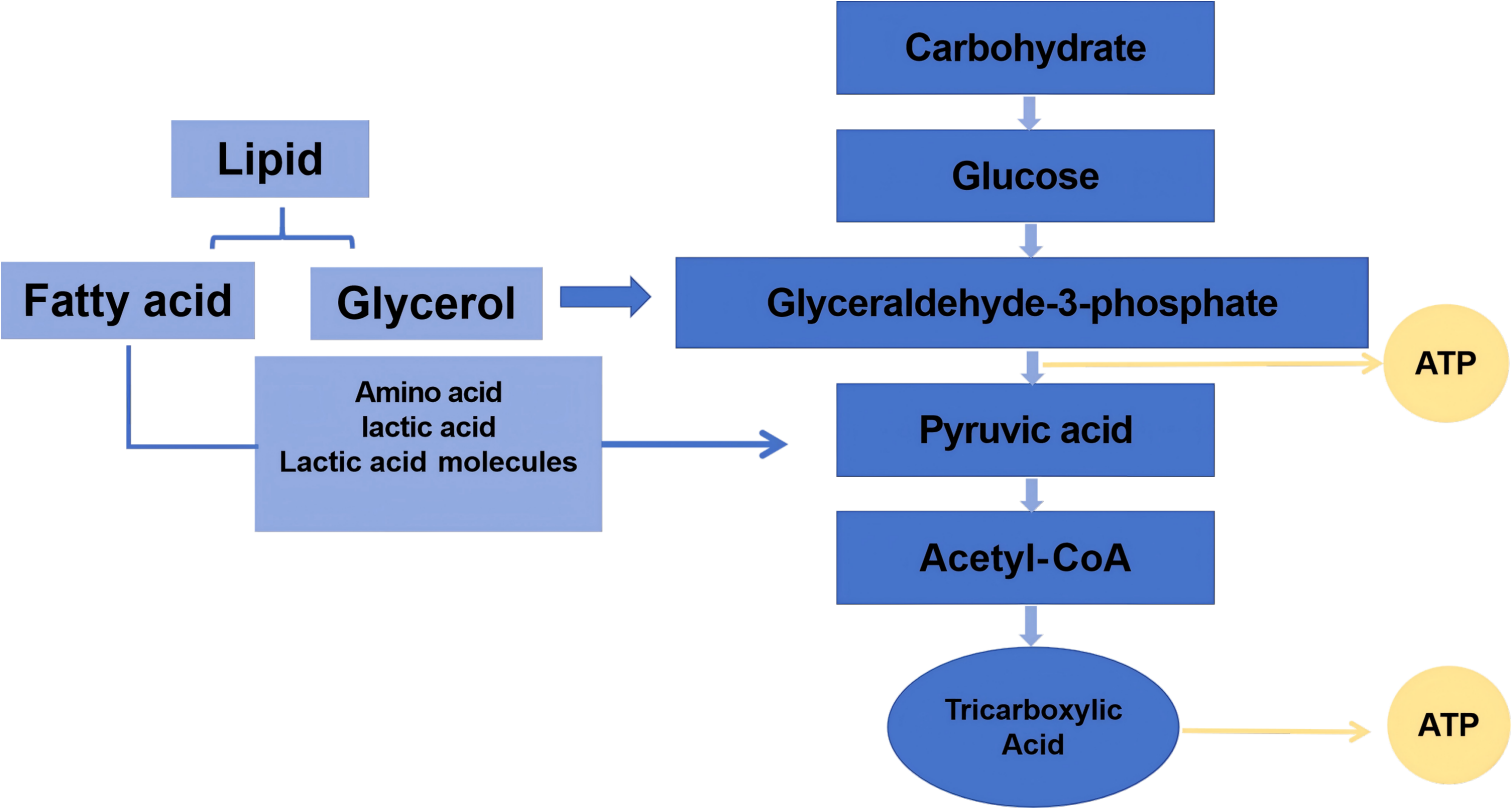
The energy in the process of exercise.

The initial energy demands during exercise are primarily met through the rapid depletion of high-energy phosphagens, notably adenosine triphosphate (ATP) and creatine phosphate (CP), collectively termed the phosphagen system (Fig. 2). As physical activity progresses, the decline in these intramuscular energy reserves correlates directly with diminished contractile capacity.

High-energy phosphate compounds central to exercise metabolism include ATP and phosphocreatine (creatine phosphate, PCr). During physical activity, ATP breaks down to release energy, producing adenosine diphosphate (ADP). Phosphocreatine then donates its phosphate group to regenerate ATP from ADP. Concurrently, two ADP molecules can combine *via* the enzyme myokinase to form one ATP and adenosine monophosphate (AMP), releasing inorganic phosphate ions (Pi) (Hackney, 2016). Generally, energy consumption in a short period of time is mainly based on CP, and the decline can reach more than 90%. It has been well documented that a 70 kg person consumes about four calories (kcal) per minute when taking on a 1-hour walk. As exercise time increases, the calories consumed gradually increase (Thompson et al., 2013). Therefore, ATP and CP and their related metabolites, AMP, ADP, and Pi, can be used as one of the biomarkers for the preliminary assessment of exercise-induced fatigue.

#### Glucose and glycogen

During prolonged strenuous exercise, the main energy substance consumed is saccharides. After long exercise, glucose in the blood is consumed. With further extension of exercise duration, muscle glycogen and liver glycogen are greatly consumed. At this stage, up to 75–90% of muscle glycogen and more than 90% of liver glycogen stores may be depleted. Glycogen cannot maintain normal blood glucose levels and often resulting in hypoglycemia (Hackney, 2016; Nelson & Cox, 2005).

#### Fat

As exercise progresses, fat will also begin to be consumed; however exercise-induced fatigue will not lead to a large reduction of body fat (Romijn et al., 1993; Van Loon et al., 2001). Although the overall fat content does not change much, the levels of fatty acids and triglycerides in the blood will increase (Liao et al., 2024). Jie (1998) demonstrated that plasma free fatty acid concentrations in the blood during surface body exercise could rise from 0.1 mmol/L to 2 mmol/L. It is generally believed that fatigue onset can be delayed to some extent if endurance training can improve the utilization of fat during exercise, thereby reducing the consumption of glycogen and preventing a decline in blood glucose levels.

#### Amino acid related to energy metabolism

During exercise, free amino acids and intracellular amino acids in the blood are consumed and utilized. Among these, glutamine, leucine, isoleucine and valine are the amino acids currently considered to be related to energy metabolism. These amino acids in blood are consumed as exercise progresses and can also serve as biomarkers of exercise-induced fatigue (McGlory & Phillips, 2016).

Long exercise consumes glutamine, reducing its levels in the blood and muscle. The enzyme activity of glutamine is reduced due to the decrease in glycogen and blood glucose (Mioko, Yuji & Kazuto, 2013).

Valine, leucine and isoleucine are all branched-chain amino acids. These amino acids can be catabolized in muscle tissue and can be used by oxidative energy supply. After marathon runners supplemented branched chain amino acids, weariness decreased significantly. The results of comprehensive literature show that the branched chain amino acids are beneficial during short-term, moderate-intensity exercise; after prolonged exercise (>3 h) or when exercise-induced fatigue occurs, their depletion shows a high correlation with the state of fatigue.

While the depletion of energy substrates like glycogen and CP provides a logical and historically significant explanation for fatigue, their utility as real-time, predictive biomarkers is limited by several factors. Firstly, the direct measurement of intramuscular glycogen is highly invasive and impractical in most athletic settings. Secondly, there is considerable inter-individual variability in substrate utilization rates and fatigue thresholds, meaning a ‘one-size-fits-all’ critical level of depletion does not exist (Ament & Verkerke, 2009). For instance, well-trained athletes exhibit enhanced glycogen sparing and fat oxidation capabilities, delaying the point at which glycogen depletion triggers fatigue (Zafrilla, Marhuenda & Arcusa, 2019). Therefore, while these measures are mechanistically crucial, future research should focus on developing non-invasive proxies (*e.g.*, *via* breath or sweat analysis) or dynamic metabolic models that can predict individual substrate depletion kinetics, rather than relying on static, *post-hoc* measurements.

### Metabolite

During exercise, energy metabolism becomes exceptionally vigorous, leading the body to generate various metabolic substances. As these metabolites accumulate, they will contribute to a decline in the body’s exercise capacity, thereby leading to the emergence of exercise-induced fatigue. Common metabolites observed during exercise include lactate, ammonia, urea, ketone bodies, and others. These metabolites are frequently employed as biomarkers for indicating exercise-induced fatigue. Specifically, lactateis produced *via* anaerobic glycolysis when the body’s oxygen supply is insufficient during intense exercise. Ammonia is a byproduct of protein metabolism, and its accumulation can affect the normal functioning of the nervous system. Urea is a waste product of protein metabolism that needs to be excreted from the body. The presence of ketone bodies indicates that the body is using fat as an energy source, which may occur during prolonged exercise or when carbohydrate reserves are depleted. These metabolites play a crucial role in understanding the physiological changes that occur during exercise and in diagnosing and monitoring exercise-induced fatigue (Khadartsev, Fudin & Badtieva, 2022).

#### Lactic acid

The human body consumes a lot of ATP and CP during intense exercise. When these substrates become insufficient, the body begins to use the lactic acid system (anaerobic glycolysis) for energy supply in a short duration. During this process, ATP is produced by glucose under anaerobic conditions. Due to the low efficiency of ATP production *via* anaerobic glycolysis, the body will conduct a large amount of anaerobic glycolysis in order to produce enough energy, producing large amounts of lactic acid. Notably, this rise in blood lactate concentration is primarily driven by muscle contraction demands, with a significant contribution from the recruitment of fast-twitch muscle fibers (Sánchez-Medina & González-Badillo, 2011). These fibers are characterized by high force output but low fatigue resistance, and their heightened expression of glycolytic enzymes (*e.g.*, phosphofructokinase, lactate dehydrogenase) accelerates glycogen breakdown. This causes pyruvate production rates to exceed the oxidative capacity of the mitochondria, leading to disproportionately high lactate generation compared to slow-twitch fibers (Garcia-Sillero et al., 2022). Yan et al. (2022) found through bicycle experiments in the 80s that exercise-induced fatigue was associated with elevated lactic acid after exercise.

Lactic acid itself does not cause fatigue; rather fatigue is associated with the H+ dissociated from lactic acid. The decrease in pH affects many processes, including the ability of troponin to bind calcium, as well as the activity of many enzymes. Previous studies have confirmed that the pH value decreases, which reduces the activity of kinases such as creatine kinase, ATPase, and phosphofructokinase (PFK), thereby affecting the metabolism of the lactate system (McCown, Reibel & Micozzi, 2010). This mechanism of lactate production, dominated by fast-twitch fiber recruitment, explains the rapid surge in blood lactate observed during high-intensity exercise (Garcia-Sillero et al., 2022). Consequently, lactate and the associated pH decrease remain established biomarkers for assessing exercise-induced fatigue and exhaustive exertion.

#### Ammonia

Studies have confirmed that during long-term high-intensity exercise, proteins and amino acids in the human body are consumed to participate in the energy supply. Due to the decomposition of proteins during long-term exercise, amino acid decomposition produces ammonia (Khadartsev, Fudin & Badtieva, 2022). Studies have shown that the increase in ammonia concentration plays an important role in both central and peripheral fatigue (Meeusen et al., 2006). In general, high concentrations of ammonia can affect ATP synthesis. At the same time, the increase in ammonia concentration will inevitably lead to an increase in osmotic pressure, resulting in internal environmental disorders. In addition, ammonia can enter the brain tissue through the blood–brain barrier, which has a toxic effect on the brain and affects the function of the central nervous syste. Current research suggests that ammonia hinders the synthesis of inhibitory GABA (*γ*-aminobutyric acid). Due to GABA deficiency, nerve control is reduced, resulting in fatigue (Foley et al., 2006).

#### Urea

Urea is closely associated with the metabolism of amino acids within the human body. Consequently, it enables us to assess the physical functioning and fatigue levels of athletes in a more comprehensive manner. It is widely acknowledged that the quantity of blood urea tends to rise in proportion to the exercise load, and its recovery process is relatively sluggish. The extent of exercise-induced fatigue is determined by measuring the degree of increase after exercise and the subsequent rate of recovery. When the level of urea in the blood following exercise is three mmol/L higher than that before exercise, it can be construed as an indication of a substantial amount of exercise, signifying that the athlete has reached the fatigue threshold (Qian et al., 2024).

Currently, urine and saliva are widely used as non-invasive biomarkers for fatigue monitoring in sports. To improve their measurement accuracy, it is essential to overcome the challenges in standardizing sample processing and advancing detection technologies. The key steps include: first, sample collection and storage processes must be strictly standardized. For urine, analysis must be conducted within 2 h (or within 3 h if refrigerated) to prevent biomarker degradation. For saliva, the addition of RNA stabilizers (such as RNAlater) enables preservation at room temperature for up to one year, significantly reducing the risk of biomarker degradation caused by repeated freeze-thaw cycles (Zheng et al., 2025).

Secondly, high-sensitivity detection technologies were applied. Automatic online solid-phase microextraction coupled with liquid chromatography-tandem mass spectrometry (SPME-LC/MS/MS) achieved a precision of 4.9% for cortisol detection in 40 µL of saliva (quantification limit: 0.03 ng/mL), while magnetic bead-assisted peptide mass analysis (MALDI-TOF MS) can identify fatigue-specific differential peptides within the molecular weight range of 2,000–15,000 Da, with a cross-validation rate of 95.49%, providing new targets for the development of portable devices (Gervasoni et al., 2018). Further elimination of systematic errors through multimodal data integration: combining salivary cortisol/*α*-amylase with urine urea/uric acid ratios and integrating psychological scales (such as POMS) to establish a machine learning assessment model, reducing the overall error rate by 32%. Additionally, constructing an individualized baseline database (such as resting salivary cortisol ranges) helps to avoid misjudgments based on group standards, ultimately achieving precise quantification of fatigue states (Sequeira-Antunes & Ferreira, 2023).

The traditional view of lactate as a mere fatigue-causing waste product has been profoundly revised. The ‘lactate shuttle’ hypothesis re-frames lactate as a crucial energy carrier and signaling molecule (Brooks, 2018). This paradigm shift challenges the oversimplified acidosis model, as the relationship between pH decline and fatigue is not always direct or causal (Westerblad, Allen & Lännergren, 2002). Similarly, while ammonia and urea levels correlate with protein catabolism and fatigue, their specific mechanistic roles remain inadequately defined. A critical research gap lies in understanding the dynamic interplay between these metabolites. Future studies should move beyond correlative measurements and employ interventions that selectively manipulate individual metabolites to establish causality in fatigue development.

### Metabolic kinases and products in the blood

During exercise, blood assumes a crucial transportation role, delivering oxygen to meet muscular energy demands and conveying essential glucose. Metabolites like lactate and ammonia directly reflect energy substrate turnover during exercise. Simultaneously, circulating enzymes and signaling molecules in the bloodstream provide critical insights into cellular stress responses and systemic metabolic regulation under fatigue conditions. These biomarkers—including kinases, redox mediators, and endocrine factors—serve dual roles: as functional indicators by quantifying energy flux (*e.g.*, ATP regeneration), oxygen dynamics, and mitochondrial efficiency; and as damage signals revealing membrane integrity loss, oxidative injury, or hormonal dysregulation induced by exercise. This section examines key blood-based mediators whose fluctuations correlate strongly with exercise-induced fatigue, spanning energy buffering systems (CK), oxygen transport machinery (Hb), vascular regulators (NO/NOS), mitochondrial enzymes (SDH), and anabolic-catabolic balance (T/C ratio).

#### Creatine kinase

Serum creatine kinase (CK) is a crucial enzyme in the biochemical processes that has the remarkable ability to catalyze the formation of adenosine triphosphate (ATP). primarily through the CK/phosphocreatine (PCr) system. This system minimizes [ATP]/[ADP] fluctuations during high-intensity activities to sustain contractile function (Dahlstedt et al., 2003). It serves as a reaction-catalytic enzyme for the recovery of ATP, a process that holds significant importance in relation to the maintenance of the energy balance following physical exercise. The presence of serum creatine kinase is primarily attributed to the movement of creatine kinase from within the muscle cells into the serum across the cell membranes. Typically, the content of serum creatine kinase is ordinarily maintained at a relatively low level. However, when the body undergoes exercise-induced fatigue, it leads to an increase in the permeability of the cell membranes. As a result, creatine kinase is liberated from the cells and enters the bloodstream, thereby causing a notable elevation of the serum creatine kinase content (Qian et al., 2024; Shijing et al., 2016).

Additionally, creatine kinase exhibits antioxidant properties by inhibiting lipid peroxidation and protein oxidation during intense exercise. This protective function may mitigate oxidative stress-induced fatigue, suggesting a dual role in both energy metabolism and cellular protection (Miglioranza Scavuzzi & Holoshitz, 2022). In acute fatigue scenarios, changes in neuromuscular parameters (*e.g.*, reduced peak power, prolonged contraction time) correlate more strongly with performance decline than creatine kinase elevation, indicating CK’s limited sensitivity as a real-time fatigue biomarker during short-term exhaustive exercise (Yánez, 2023).

#### Hemoglobin

Hemoglobin, the main component of red blood cells, plays a crucial role in delivering oxygen and carbon dioxide. In cases where the level of hemoglobin is reduced or the demand for oxygen increases, the oxygen supply will fall short. This shortage will subsequently lead to a decrease in exercise capacity (Yin et al., 2024). During intense physical activity, exercise-induced fatigue can occur, which may cause damage to red blood cells. As a result, hemoglobin will be released from these damaged cells. This release can lead to a situation where the level of hemoglobin drops below the normal range (Shijing et al., 2016).

#### Nitric oxide and nitric oxide synthase

Nitric oxide (NO) in the body is enzymatically synthesized by nitric oxide synthase (NOS). Nitric oxide plays a crucial role in promoting the augmentation of blood flow and the dilation of blood vessels, thereby regulating the blood supply within the body (Draghici et al., 2024). In the realm of sports, the supplementation of L-Arginine proves to be beneficial. It helps in reducing the muscle injury that athletes may encounter and also contributes to the enhancement of their performance (Lomonosova et al., 2014). This is particularly significant as it directly impacts the athletes’ ability to perform at their best and minimize the risk of injury that could potentially hamper their progress and career (Jackman et al., 2010). Therefore, the decline in nitric oxide can cause exercise-induced fatigue.

#### Succinate dehydrogenase

Succinate dehydrogenase (SDH) is a key enzyme in the tricarboxylic acid cycle. Its activity can be used to assess the aerobic oxidation capacity of athletes (Lewis et al., 2010). Succinate dehydrogenase is located on the inner mitochondrial membrane and does not enter the tissue fluid and thus into the bloodstream. Due to exercise-induced muscle tissue damage, the increased permeability of the mitochondria leads to an increase in the content of succinate dehydrogenase in the coating slurry, which can be used to reflect the status of the tricarboxylic acid cycle (Rodrigues et al., 2010).

#### Testosterone/cortisol

Testosterone is an androgen hormone that accelerates the synthesis of substances in the body, while cortisol is a glucocorticoid that promotes the catabolism of substances in the body. The testosterone/cortisol (T/C) ratio serves as an indicator of the anabolic and catabolic balance of nutrients in the body. Unlike free testosterone concentrations that significantly decrease when the body is depleted due to movement, increased cortisol and its receptors cause protein breakdown beyond synthesis levels (Meeusen, 2014; Shimomura et al., 2009).

Blood-based biomarkers such as CK and hemoglobin are widely used yet frequently misinterpreted. A major limitation is their lack of specificity for fatigue. For instance, elevated CK levels serve as a robust indicator of muscle damage but do not directly reflect the acute state of fatigue that impairs performance within a single exercise bout (Brancaccio, Maffulli & Limongelli, 2007). Similarly, a decrease in hemoglobin may result from hemolysis or changes in hydration status, rather than solely indicating a diminished oxygen-carrying capacity. The T/C ratio, while valuable for assessing long-term anabolic-catabolic balance, is affected by diurnal rhythms, nutrition, and psychological stress, thereby complicating its interpretation in the context of acute fatigue (Küüsmaa, Sedliak & Häkkinen, 2015; Vaamonde et al., 2022). Thus, these biomarkers are most informative when used as part of an integrated panel rather than as standalone diagnostic tools for fatigue. Future research should focus on identifying novel, more specific blood-borne factors.

### Free-radica-associated biomarkers

Beyond endocrine regulation, intense exercise triggers a cascade of oxidative reactions. When oxygen consumption surges during high-intensity exertion, reactive oxygen species (ROS)—unstable molecules with unpaired electrons—are overproduced through mitochondrial electron leakage and neutrophil activation. These radicals initiate chain reactions by “stealing” electrons from lipids, proteins, and DNA, a process that culminates in oxidative stress when ROS production exceeds the body’s endogenousantioxidant capacity. This imbalance manifests primarily as lipid peroxidation (measured by Malondialdehyde) and antioxidant enzyme adaptation (super oxide dismutase, catalase, glutathione peroxidase). Collectively, these biomarkers quantify exercise-induced macromolecular damage and compensatory defenses (Hackney, 2016).

#### Malondialdehyde

Malondialdehyde (MDA) is a product of degradation by peroxidation *in vivo*. To some extent, its concentrarion can reflect the severity of free radical attack and damage to motor cells. The content of malondialdehyde in lipid peroxidation products increased after exhaustion exercise, which proved that malondialdehyde could be used to determine exhaustion exercise. Mitchell’s post-run plasma analysis of ultra-long marathon runners confirmed that malondialdehyde levels in body were significantly elevated after exercise (Maxwell et al., 2001; Mohammadi et al., 2025).

#### Superoxide dismutase

Superoxide dismutase (SOD) is an important antioxidant enzyme in the free radical scavenging system. Its activity level can indicate the body’s free radical load. When the body has a high free radical content during exercise-induced fatigue, the high activity of superoxide dismutase is required. After prolonged exercise, the results showed a significant increase in malondialdehyde content in the plasma, as well as an increase in the activity of superoxide dismutase. By analyzing SOD/MDA, it can reflect the free radical production and clear rate in the body, and further analyze the actual changes of free radical metabolism in depth and objectively, thereby indicating the degree of exercise-induced fatigue of the body (Zhao et al., 2024).

#### Catalase

Catalase (CAT) is one of the key enzymes for scavenging intracellular H_2_O_2_. H_2_O_2_ is the reduction product of O_2_ andhas strong oxidation properties. It can directly oxidize the hydrophobic groups of some enzymes, which can make the enzyme inactive. Catalase can bind to and clear the hydrogen peroxide *in vivo*. Quintanilha found that catalase activity increased in skeletal and cardiac muscle of rat after aerobic endurance training, indicating that catalase activity in muscle can be improved as exercise progressed (Lew & Quintanilha, 1991). The activity of catalase in the human body is highly sensitive to exercise stimulation. Aerobic exercise can significantly promote the increase of catalase activity in the body, and if the exercise intensity increases, the catalase activity will be further increased (Ekström et al., 2025).

#### Glutathione peroxidase

Glutathione peroxidase (GSH-PX) is an enzyme that catalyzes the reduction reaction of H2O2, thus protecting membrane structural integrity. Most studies suggest that exercise-induced fatigue lesds to elevated glutathione peroxidase activity. Heavy exercise can cause a significant increase in glutathione peroxidase activity in muscle tissue. Lew et al. reported that when rats ran to exhaustion, glutathione peroxidase, glutathione S-transferase, and glutathione reductase activities increased in the liver and bone, while their activities decreased in plasma (Lew, Pyke & Quintanilha, 1985; Wang et al., 2024; Wu, Liu & Chen, 1999).

The role of oxidative stress in fatigue is a field of significant debate. The ‘mitohormesis’ theory posits that moderate ROS production is essential for adaptive signaling and training responses (Ristow & Zarse, 2010). Therefore, simply observing an increase in MDA or antioxidant enzyme activity does not necessarily indicate deleterious fatigue; it could signify a positive adaptive process. A majority of studies measure these markers in blood, but their levels may poorly reflect the redox environment within contracting muscle fibers (Powers, Radak & Ji, 2016). Furthermore, the inconsistent outcomes of antioxidant supplementation studies in combating fatigue challenge the simplistic ‘oxidants are bad’ narrative (Merry & Ristow, 2016). Future research should focus on the targeted measurement of redox status in specific cellular compartments and during recovery to determine whether oxidative stress is a cause, a correlate, or a consequence of exercise-induced fatigue.

### Central neurotransmitter-related biomarkers

Extensive research conducted over several years has consistently demonstrated that neurotransmitters within the central nervous system (CNS) play a crucial role in motor fatigue, with particular significance for central fatigue. These studies have established that specific substances, including serotonin, norepinephrine, dopamine, acetylcholine, amino acids, and other compounds, are critically involved in the transmission processes underlying exercise-induced fatigue. Serotonin regulates various physiological pathways contributing to fatigue, while norepinephrine influences stress responses and energy expenditure, both impacting fatigue levels. Dopamine, central to motivation and reward systems, can become dysregulated associated with fatigue development. Acetylcholine is essential for neuromuscular communication, and its dysfunction can directly lead to muscle fatigue. In addition, amino acids and other identified compounds play distinct roles in the complex mechanisms of exercise fatigue.

#### Hydroxytryptamine

Hydroxytryptamine (5-HT) is a metabolite of tryptophan and a crucial neurotransmitter in the central nervous system, involved in various physiological roles. Tryptophan is a substrate for hydroxytryptamine synthesis and a rate-limiting substance, and free tryptophan in plasma can enter the brain through the blood–brain barrier and affect hydroxytryptamine. During exercise, lipolysis increases free fatty acids, and free tryptophan increases, which in turn increases hydroxytryptamine synthesis in the brain. Studies have shown that exercise can increase hydroxytryptamine levels in the central system, and this increase is associated with the development of central fatigue. Newsholme, Gordon & Newsholme (1987) first proposed that hydroxytryptamine may be a regulator of central fatigue. Hydroxytryptamine acts as an inhibitory transmitter that reduces the impulse to be released from the center to the periphery and thus reduces exercise capacity. Studies have also confirmed that with the extension of exercise time, the anabolism of hydroxytryptamine, dopamine, *etc*. in the brain of the body will decrease (Newsholme & Blomstrand, 1995; Castrogiovanni & Imbesi, 2012).

#### Dopamine (DA)

Dopamine can regulate the tension degree of the muscle tissue, and dopamine was the first neurotransmitter confirmed to play an important role in exercise-induced fatigue (Liao et al., 2024). Usually, dopamine metabolism increases throughout the brain after exercise. Sutoo Allen, Lamb & Westerblad (2008) has found that there are two main reasons for the increase in dopamine in the brain after initial exercise, one is to promote the synthesis of dopamine, and the other is to promote the binding of dopamine receptors. However, studies have shown that the synthesis of dopamine in the rat midbrain is weakened during central fatigue, and that a high dopamine level can delay fatigue development (Kanter et al., 1985; Lu et al., 2024).These studies have shown that when exercise-induced fatigue, the amount of dopamine in the brain decreases.

#### Noradrenaline

Noradrenaline (NE) is a neurotransmitter synthesized and secreted by adrenergic nerve terminals. It is produced from dopamine through catalysis by the enzyme dopamine *β*-hydroxylase. Studies have confirmed that noradrenaline in the hypothalamus decreases after exercise and exhaustion, and the content of noradrenaline can affect the metabolic level of noradrenalin, both of which can inhibit the normal effect of the hypothalamus (Hackney, 2016; Lew, Pyke & Quintanilha, 1985), which is one of the causes of exercise-induced fatigue.

#### Acetylcholine

Acetylcholine (Ach) is a cholinergic neurotransmitter released within the central nervous system by cholinergic nerve endings. Its synthesis and release are vital for central nervous system. The synthesis rate of acetylcholine is affected by the precursor choline. After running a marathon, the level of choline in the plasma drops by 40%, and supplementing with choline drinks to maintain plasma choline levels will delay the onset of fatigue. Supplementing with choline drinks during marathons can delay the onset of fatigue (Fecik et al., 2024).

#### Amino acid (neurotransmitter correlation)

At present, it is believed that the amino acids related to central fatigue mainly include gamma-aminobutyric acid (GABA), glutamic acid (Glu), and branched-chain amino acids (BCAA). BCAAs, which include isoleucine, leucine, and valine, are important for energy supply. As discussed earlier, the role of BCAAs in fatigue has been introduced (Roelands et al., 2009).

Gamma-aminobutyric acid is an inhibitory neurotransmitter. One cause of central motor fatigue is an increase in gamma-aminobutyric acid levels. With the extension of exercise time, the body will appear hypoxia, making the gamma-aminobutyric acid oxidation process weakened, and the high concentration of gamma-aminobutyric acid will cause postsynaptic inhibition (Sutoo & Akiyama, 2003). Elevated levels of gamma-aminobutyric acid in the brain can lead to exercise-induced fatigue.

Glutamic acid is a neurotransmitter related to excitability in the central nervous system, which is very abundant in the brain, and normal levels of glutamic acid play an important role in maintaining neuronal excitability, and glutamic acid is the transmitter of most excitatory synapses. When the amount of glutamic acid in the brain changes abnormally, it leads to a decline in the function of the central system, which is onecause of exercise-induced fatigue (Li et al., 2020).

#### Tissue endothelin

NO can promote vasodilation and is also an important neurotransmitter, which has an important physiological role in exercise, especially in the cardiovascular system. NO is mainly manifested in tissue cells as intracellular messenger molecules, which can cause vascular smooth muscle relaxation through the cGMP interaction. Studies have confirmed that normally NOS is functionally active in brain tissue, and the expression of NOS can be significantly weakened after heavy-load exercise training, indicating that fatigue can reduce NOS expression in brain tissue (Anish, 2005).

ET is an active small molecule polypeptide that promotes vasoconstriction, and its constrictive effect on blood vessels is the most effective substance, contrary to the effect of NO. Previous studies have confirmed that exercise promotes the enhancement of tissue endothelin (ET) expression, causing vasoconstriction, which in turn leads to hypoxia in the body. The expression of tissue endothelin is highly correlated with exercise intensity, and exercise-induced fatigue occurs due to ischemia and hypoxia when the exercise load is too large (Meeusen et al., 2007).

A key limitation in this field remains the overreliance on peripheral measures to infer central neurotransmitter changes. Since the blood–brain barrier restricts free exchange of molecules between the periphery and the brain, such inferences are highly speculative (Meeusen et al., 2007). The classic “central fatigue hypothesis,” once centered on hydroxytryptamine, has been challenged: dopamine and noradrenaline are now considered equally important in regulating motivation and perceived exertion (Roelands & Meeusen, 2010). Methodological constraints also present a challenge. While animal studies permit direct brain measurement *via* techniques like microdialysis, human studies rely on indirect proxies. Notably, interactions between peripheral metabolites and central neurotransmission form a promising yet underexplored research frontier.

### Biomarkers in the urine

By meticulously measuring the concentration of metabolites in the urine using advanced analytical techniques, it is possible to indirectly mirror the metabolic changes occurring in the body. This, in turn, enables us to infer the extent of exercise-induced fatigue. The analysis of urine metabolites provides valuable insights into the body’s response to physical exertion and helps in understanding the mechanisms underlying exercise-induced fatigue (Thompson et al., 2013). Moreover, urinalysis is of great significance in clinical and athletic body evaluation. Biomarkers in urine are one of the reliable indicators for detecting exercise-induced fatigue (Table 1).

**Table 1:** Biomarkers associated with exercise fatigue in routine urine testing.

**Biomarkers**	**Source**	**Normal condition**	**Exercise fatigue**	**Reference**
Urine PH	Lactic acid, pyruvate,etc	PH=7	PH drops by 3–4	Chiron et al. (2024); Kondoh, Kawase & Ohmori (1992)
Urine protein	Glomerular filtration	Microscale 2–8 mg	Increase by 3–4 times	Park & Moon (2022)
Urinary occult blood	Red blood cell filtration	Can’t detect	Can be detect	Zhang et al. (2025)
Bilirubin	Hemoglobin breakdown	Normal value	Rise	Coso et al. (2013)
Urobilinogen	Hemoglobin breakdown	Normal value	Rise	Yamagata & Sakuraba (2019)
Urine creatinine	Creatine or CP breakdown	Normal value	Rise	Corsetti et al. (2016)

### Saliva

Saliva is a highly convenient, completely safe, and non-invasive characteristic for collection. In recent years, the research focused on the utilization of saliva to gauge the exercise status has been drawing an increasing amount of attention. This is particularly true in the field of sports. For athletes, the option of using saliva instead of blood and urine to evaluate the body’s athletic condition presents itself as a significantly faster and more convenient approach. The potential of saliva as a source of biomarker for assessing exercise-induced physiological changes is being explored in numerous studies. Its collection is not only less invasive but also more readily accepted by athletes, thereby reducing the discomfort and potential risks associated with traditional methods. Moreover, the analysis of saliva can provide valuable insights into various aspects of an athlete’s physical condition, such as hormone levels, immune function, and oxidative stress. This emerging field of research holds great promise for improving the monitoring and management of athletes’ health and performance. In the future, further advancements in salivary biomarker research are expected to lead to more accurate and comprehensive assessments of the body’s response to exercise, enabling athletes and coaches to make more informed decisions regarding training and competition (Hackney, 2016).

#### Saliva PH

Due to the increase in lactic acid production after long-term strenuous exercise, the amount of CO2 in the blood increases, and acidic substances such as ketone bodies and pyruvate accumulate, resulting in a decrease in the pH value of blood and thus a decrease in the pH value of saliva (Grzesiak-Gasek & Kaczmarek, 2022). When exercise-induced fatigue, acidosis often occurs, acidosis can reduce the body’s muscle exercise capacity and also cause symptoms of central nervous system fatigue. Adequate alkaline substances should be supplemented after exercise.

#### Salivary immunoglobulin A

Salivary immunoglobulin A (SIgA) level is one of the important indicators of human immune status. After intensive exercise or long and intense exercise, the immunosuppression caused by exercise leads to a decrease in immunoglobulin A level. Most research indicates that s-IgA level can serve as an indicator for evaluating exercise load, as it decreases following short periods of intense exhaustive exercise. Fahlman et al. (2001) performed 30 s full anaerobic work test for 3 min, which indicated that a temporary decrease in saliva immunoglobulin A levels in women. In summary, saliva immunoglobulin A is a valuable biomarker for assessing exercise-induced fatigue.

#### Other components in the saliva were used as biomarkers

With the development of high-throughput technology and their application in athletics, more components are identified in saliva, and they can replace the corresponding detection in the blood. These components are also markers of exercise activity. Guo et al. (2010) used the serum and saliva of athletes before and after exercise as the research object, and found the sports-related biochemical indicators in saliva. Giles et al. (2012) identified a class of small molecule proteins (sMW) in saliva after exercise through high-throughput proteome combined chromatography and mass spectrometry, and then correlated the fatigue degree of each peptide exercise, and obtained a positive correlation between a class of small molecule proteins and exercise-induced fatigue.

Enthusiasm for non-invasive biomarkers must be tempered by a critical awareness of their pre-analytical and analytical vulnerabilities. Hydration status significantly influences urinary analyte concentration, necessitating creatinine correction and rigorous sampling protocols (Cone et al., 2009). Similarly, salivary measurements of hormones such as cortisol and IgA are exquisitely sensitive to circadian rhythms, sampling techniques (stimulated *vs.* unstimulated), and oral health status (Grzesiak-Gasek & Kaczmarek, 2022). A key limitation is that many studies report changes in these biomarkers without first establishing validated, population-specific reference ranges under exercise conditions. Consequently, although non-invasive biomarkers are well-suited for longitudinal monitoring, their current utility for cross-sectional or single-time-point diagnosis of fatigue remains limited. The future direction lies in developing integrated sensor systems for real-time biomarker measurement, combined with machine learning methods to interpret the complex, multi-parameter data they generate.

## Detection Methods for Biomarkers

### Development of blood testing technologies

**Figure 3:**
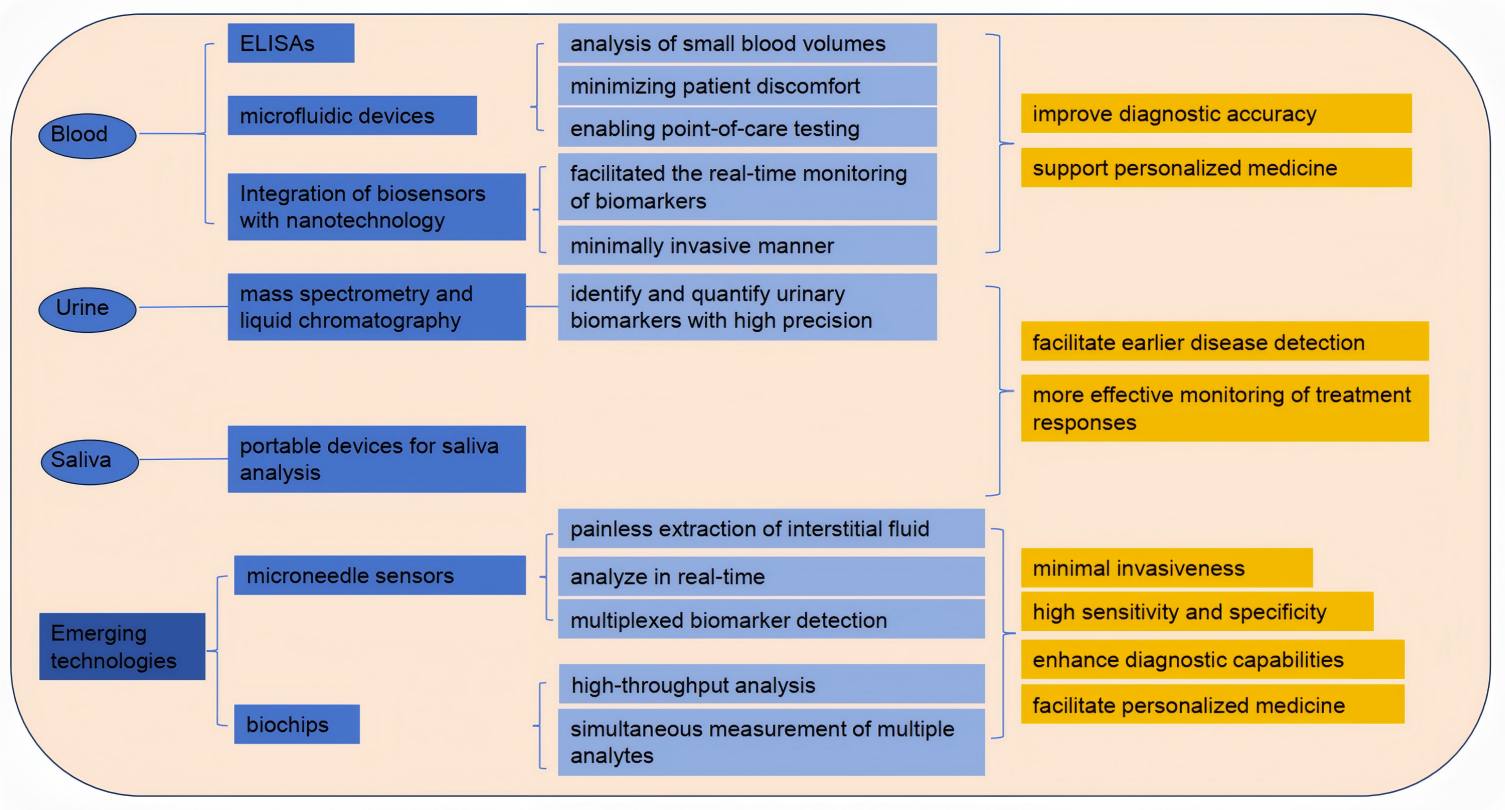
Detection of exercise fatigue related biomarkers.

The evolution of blood testing technologies has significantly enhanced the detection and monitoring of biomarkers for various diseases. Traditional methods, such as enzyme-linked immunosorbent assays (ELISAs) and blood routine tests, have paved the way for more advanced techniques. Blood routine testing is widely employed not only in clinical practice but also for assessing athletes’ physical condition, providing deeper insights into the relationships among blood biomarkers, exercise performance, and exercise-induced fatigue. Recent innovations include microfluidic devices that enable the analysis of small blood volumes, thus minimizing patient discomfort and enabling point-of-care testing (POCT) (Kim et al., 2022). Moreover, the integration of biosensors with nanotechnology has facilitated the real-time monitoring of biomarkers such as glucose and lactate, in a minimally invasive manner (Hu et al., 2024). These advancements not only improve diagnostic accuracy but also support personalized medicine by enabling tailored treatment strategies based on individual biomarker profiles. Furthermore, the development of portable devices for blood analysis has the potential to revolutionize healthcare delivery, particularly in remote or under-resourced areas (Hu, Yang & Luo, 2022). Nanotechnology-integrated biosensors facilitate real-time detection of critical fatigue markers: Monash’s 5-biomarker nano-sensor identifies 24-hour sleep deprivation with >99% accuracy for drowsy driving legislation, while subcutaneous microneedle arrays synchronize glucose/urea dynamics with ecological momentary assessment (EMA) to pinpoint dialysis-induced hypoglycemia as a modifiable fatigue trigger (Brys et al., 2021). These portable systems overcome geographical barriers—battlefield TRP/BCAA ratio detection *via* paper-based microfluidics reduces accident rates by 29% through targeted nutritional intervention (Pollock et al., 2012). As research continues to explore novel biomarker candidates and detection methods, the landscape of blood testing technologies is poised for further transformation, ultimately leading to improved patient outcomes (Fig. 3).

### Analysis of biomarkers in urine and saliva

Urine and saliva have emerged as valuable non-invasive biofluids for biomarker analysis, offering significant advantages over traditional blood sampling methods. Urine is particularly advantageous due to its ease of collection and the presence of a wide range of biomarkers indicative of various diseases, including kidney dysfunction and metabolic disorders (Meng et al., 2023). Recent studies have employed advanced techniques, such as mass spectrometry and liquid chromatography, to identify and quantify urinary biomarkers with high precision (Xu et al., 2023). Similarly, saliva has gained attention for its potential to reflect systemic health, with numerous studies highlighting the presence of biomarkers related to oral health, systemic diseases, and even cancer (Eftekhari et al., 2022). The development of portable devices for saliva analysis, coupled with advancements in proteomics and metabolomics, has enhanced the feasibility of using saliva as a diagnostic tool (Sánchez-Medina & González-Badillo, 2011).

### Emerging technologies: microneedle sensors and biochips

Microneedle sensors and biochips represent the forefront of biomarker detection technologies, combining minimal invasiveness with high sensitivity and specificity. Microneedles allow for the painless extraction of interstitial fluid, which can be analyzed for various biomarkers, including glucose, lactate, and electrolytes, in real-time (Dervisevic et al., 2024). Recent advancements in microneedle technology have led to the development of integrated biosensing platforms capable of multiplexed biomarker detection, enabling comprehensive health monitoring (Huang et al., 2023). Additionally, biochips, which utilize microfabrication techniques, have revolutionized the way biomarkers are detected by allowing for high-throughput analysis and the simultaneous measurement of multiple analytes (Zhong et al., 2024). These technologies not only enhance diagnostic capabilities but also facilitate personalized medicine by providing continuous monitoring of biomarkers in various physiological conditions. As research continues to refine these technologies and address current limitations, the application of microneedle sensors and biochips in clinical settings is expected to expand, ultimately improving patient care and outcomes (Teymourian et al., 2021).

## Future Research Directions and Challenges

### Applications of multi-omics technologies in exercise-induced fatigue research

The advent of multi-omics technologies has revolutionized the understanding of exercise-induced fatigue by integrating various biological data types, including genomics, proteomics, metabolomics, and transcriptomics. These technologies enable researchers to obtain a comprehensive view of the molecular mechanisms underlying fatigue during and after exercise. For instance, recent studies have shown that integrating transcriptomic data with metabolomic profiles can help elucidate the metabolic pathways that are altered during fatigue, providing insights into how different energy substrates are utilized by skeletal muscle (Xia et al., 2023). Furthermore, multi-omics approaches can identify novel biomarkers for fatigue, which could be instrumental in developing personalized exercise regimens tailored to individual metabolic responses. However, challenges remain in standardizing the application of these technologies, particularly regarding data integration and interpretation across different studies. Future research should focus on establishing standardized protocols for sample collection and analysis to enhance reproducibility and comparability of findings across diverse populations and exercise modalities (Ni, Sun & Li, 2022).

### Standardization and clinical applications of biomarkers

The clinical application of biomarkers for exercise-induced fatigue is hindered by a lack of standardization in their measurement and interpretation. Biomarkers such as lactate levels, heart rate variability, and cytokine profiles have shown promise in assessing fatigue, yet their clinical utility is often limited by variability in testing methods and patient populations (Sopić, Devaux & de Gonzalo-Calvo, 2024). To address these challenges, it is essential to develop standardized protocols that encompass all aspects of biomarker assessment, including pre-analytical variables, assay techniques, and interpretation frameworks (Kolev et al., 2022). Additionally, establishing clear guidelines for the clinical application of these biomarkers could facilitate their integration into routine practice, enabling healthcare professionals to monitor fatigue more effectively and tailor interventions accordingly. Future research should prioritize multi-center studies that validate the clinical relevance of fatigue biomarkers and explore their predictive capacity for exercise tolerance and recovery in various populations, including athletes and individuals with chronic diseases (Zhang, Wang & Li, 2024).

### Individual variability in exercise-induced fatigue biomarkers

Understanding the individual differences in exercise-induced fatigue is crucial for developing personalized approaches to training and recovery. Recent studies have highlighted significant variability in muscle fatigue tolerance and recovery rates among individuals, influenced by genetic, physiological, and psychological factors (Holgado et al., 2023). This variability suggests that a one-size-fits-all approach to exercise may not be effective for all individuals, emphasizing the need for personalized training programs that consider these differences. Future research should focus on identifying specific biomarkers that correlate with individual fatigue responses, potentially through the use of wearable technology and real-time monitoring systems that assess physiological parameters during exercise (Li et al., 2022). To effectively assess an individual user’s fatigue state, the use of the Rating of Perceived Exertion (RPE) scale is a practical and scientifically sound core solution. Its key advantages include ease of use, real-time feedback, and robust evidence-based support. RPE values integrate central nervous system and muscle metabolic signals, not only reflecting an individual’s current fatigue level but also enabling precise intervention by identifying personalized “fatigue points,” thereby mitigating the risk of overtraining. By widely applying RPE as a foundational tool in scenarios such as sports training and occupational health, and by integrating wearable device data in the future to establish a “subjective + objective” dual-track verification system, the precision of individualized fatigue management can be further enhanced (Zhao et al., 2025). Additionally, exploring the interplay between psychological factors, such as motivation and mental fatigue, and physical performance could provide deeper insights into the mechanisms of exercise-induced fatigue and recovery. By addressing these individual differences, researchers can enhance the effectiveness of interventions aimed at mitigating exercise-induced fatigue and improving overall athletic performance (Royer et al., 2024).

## Conclusions and Outlook

This review has synthesized current understandings of biomarkers associated with exercise-induced fatigue, covering energy substrates, metabolic byproducts, blood biochemical indicators, oxidative stress parameters, neurotransmitters, and non-invasive biomarkers. While these markers collectively shed light on the multifaceted nature of fatigue, the field remains limited by a predominance of correlative findings and fragmented insights. Moving toward a more integrated, dynamic, and causally informed understanding of fatigue mechanisms is imperative for further scientific and practical progress.

Substantial evidence supports the roles of classical biomarkers—such as lactate, ammonia, creatine kinase, and cortisol—in the study of exercise-induced fatigue. However, their interpretation is often challenging due to limited specificity, high contextual variability, and complex interactions between peripheral and central mechanisms. Meanwhile, non-invasive biomarkers from saliva and urine offer promising avenues for real-time monitoring, yet their application is hindered by insufficient standardization and validation within athletic populations. Similarly, emerging technologies like microneedle sensors and multi-omics platforms show great potential, but translating these tools into sport-specific settings requires further methodological refinement and integrated data analysis.

Looking ahead, research should prioritize elucidating the temporal dynamics and causal relationships underlying biomarker fluctuations. This will require leveraging interventional study designs and advanced computational models to decode complex, system-wide physiological networks. There is also a critical need to harmonize laboratory-based measures with field-based monitoring tools, while accounting for individual variability in baseline values and biomarker responses. Furthermore, integrating physiological indicators with perceptual and cognitive dimensions of fatigue will be essential to develop comprehensive, multidimensional assessment frameworks.

In summary, the continued advancement of research on exercise-induced fatigue depends on the adoption of integrative approaches that combine multidimensional biomarker systems, advanced technologies, and principles of personalized medicine. By addressing existing methodological limitations and prioritizing mechanistic clarity, the field can develop more effective strategies to monitor, mitigate, and manage fatigue—thereby enhancing human performance and well-being in both athletic and general populations.

## Additional Information and Declarations

### Data Availability

The following information was supplied regarding data availability: This is a literature review.
